# Epigenetic timing effects on child developmental outcomes: a longitudinal meta-regression of findings from the Pregnancy And Childhood Epigenetics Consortium

**DOI:** 10.1186/s13073-025-01451-7

**Published:** 2025-04-14

**Authors:** Alexander Neumann, Sara Sammallahti, Marta Cosin-Tomas, Sarah E. Reese, Matthew Suderman, Silvia Alemany, Catarina Almqvist, Sandra Andrusaityte, Syed H. Arshad, Marian J. Bakermans-Kranenburg, Lawrence Beilin, Carrie Breton, Mariona Bustamante, Darina Czamara, Dana Dabelea, Celeste Eng, Brenda Eskenazi, Bernard F. Fuemmeler, Frank D. Gilliland, Regina Grazuleviciene, Siri E. Håberg, Gunda Herberth, Nina Holland, Amy Hough, Donglei Hu, Karen Huen, Anke Hüls, Marjo-Riitta Jarvelin, Jianping Jin, Jordi Julvez, Berthold V. Koletzko, Gerard H. Koppelman, Inger Kull, Xueling Lu, Léa Maitre, Dan Mason, Erik Melén, Simon K. Merid, Peter L. Molloy, Trevor A. Mori, Rosa H. Mulder, Christian M. Page, Rebecca C. Richmond, Stefan Röder, Jason P. Ross, Laura Schellhas, Sylvain Sebert, Dean Sheppard, Harold Snieder, Anne P. Starling, Dan J. Stein, Gwen Tindula, Marinus H. van IJzendoorn, Judith Vonk, Esther Walton, Jonathan Witonsky, Cheng-Jian Xu, Ivana V. Yang, Paul D. Yousefi, Heather J. Zar, Ana C. Zenclussen, Hongmei Zhang, Henning Tiemeier, Stephanie J. London, Janine F. Felix, Charlotte Cecil

**Affiliations:** 1 Child and Adolescent Psychiatry/Psychology, Erasmus MC, University Medical Center Rotterdam, Rotterdam, Netherlands; 2 Department of Obstetrics and Gynecology, University of Helsinki, Helsinki, Finland; 3 Helsinki University Hospital, Helsinki, Finland; 4 ISGlobal, Barcelona, Spain; 5 Universitat Pompeu Fabra (UPF), Barcelona, Spain; 6 CIBER Epidemiología y Salud Pública, Madrid, Spain; 7 Clinical Research Practice, Westat, Rockville, MD USA; 8 Bristol Medical School, Bristol Medical School, Population Health Sciences, University of Bristol, Bristol, UK; 9 Psychiatric Genetics Unit, Group of Psychiatry, Mental Health and Addictions, Vall d’Hebron Research Institute (VHIR), Barcelona, Spain; 10 Department of Mental Health, Hospital Universitari Vall d’Hebron, Barcelona, Spain; 11 Biomedical Network Research Centre on Mental Health (CIBERSAM), Biomedical Network Research Centre on Mental Health (CIBERSAM), Instituto de Salud Carlos III, Madrid, Spain; 12 Department of Medical Epidemiology and Biostatistics, Karolinska Institutet, Stockholm, Sweden; 13 Department of Environmental Sciences, Vytautas Magnus University, Kaunas, Lithuania; 14 Clinical and Experimental Sciences, Faculty of Medicine, University of Southampton, Southampton, UK; 15 William James Center for Research, ISPA-Instituto Universitário, Lisbon, Portugal; 16 Medical School, Royal Perth Hospital Unit, the University of Western Australia, Perth, Australia; 17 Population and Public Health Sciences, Environmental Health, University of Southern California, Los Angeles, USA; 18 Department Genes and Environment, Max-Planck-Institute of Psychiatry, Munich, Germany; 19 Lifecourse Epidemiology of Adiposity and Diabetes (LEAD) Center, University of Colorado Anschutz Medical Campus, Aurora, USA; 20 Department of Medicine, Pulmonary, Critical Care, Allergy and Sleep, University of California, San Francisco, USA; 21 Center for Environmental Research and Community Health (CERCH), School of Public Health, University of California, Berkeley, USA; 22 Family Medicine and Population Health, School of Medicine, Virginia Commonwealth University, Richmond, USA; 23 Depatment of Population and Public Health Sciences, Keck Schools of Medicine, University of Southern California, Los Angeles, USA; 24 Centre for Fertility and Health, Norwegian Institute of Public Health, Oslo, Norway; 25 Department of Environmental Immunology, Helmholtz Centre for Environmental Research - UFZ, Leipzig, Germany; 26 Division of Environmental Health Sciences, School of Public Health, Children’S Environmental Health Laboratory, University of California, Berkeley, USA; 27 Born in Bradford, Bradford Institute for Health Research, Bradford Teaching Hospitals NHS Foundation Trust, Bradford, UK; 28 Department of Medicine, Division of General Internal Medicine, University of California, San Francisco, USA; 29 Department of Epidemiology, Rollins School of Public Health, Emory University, Atlanta, USA; 30 Gangarosa Department of Environmental Health, Rollins School of Public Health, Emory University, Atlanta, USA; 31 Department of Biostatistics and Bioinformatics, Rollins School of Public Health, Emory University, Atlanta, USA; 32 MRC Centre for Environment and Health, School of Public Health, Imperial College London, London, UK; 33 Research Unit of Population Health, Faculty of Medicine, University of Oulu, Oulu, Finland; 34 Unit of Primary Care, Oulu University Hospital, Oulu, Finland; 35 Department of Life Sciences, College of Health and Life Sciences, Brunel University London, London, UK; 36 Public Health Practice, WESTAT, Research Triangle Park, Raleigh, NC USA; 37 Clinical and Epidemiological Neuroscience (NeuroÈpia), Institut d’Investigació Sanitària Pere Virgili (IISPV), Tarragona, Spain; 38 Department of Paediatrics, Division of Metabolic and Nutritional Medicine, Hauner Children’s Hospital, LMU - Ludwig Maximilians Universitaet Muenchen, Munich, Germany; 39 Department of Pediatric Pulmonology and Pediatric Allergology, Beatrix Children’S Hospital and GRIAC Research Institute, University of Groningen, University Medical Center Groningen, Groningen, Netherlands; 40 Department of Clinical Sciences and Education, Södersjukhuset, Karolinska Institutet, Stockholm, Sweden; 41 Department of Epidemiology, University Medical Center Groningen, University of Groningen, Groningen, The Netherlands; 42 Environment and Health over the Lifecourse Program, Isglobal, Barcelona, Spain; 43 Universitat Pompeu Fabra (UPF), Barcelona, Spain; 44 CIBER Epidemiología y Salud Pública, Barcelona, Spain; 45 Department for Clinical Science and Education, Södersjukhuset, Karolinska Institutet, Stockholm, Sweden; 46 Health and Biosecurity, CSIRO, Canberra, Australia; 47 Psychiatry/Psychology, Erasmus MC, University Medical Center Rotterdam, Rotterdam, The Netherlands; 48 Department of Physical Health and Ageing, Division for Physical and Mental Health, Norwegian Institute of Public Health, Oslo, Norway; 49 Human Health, Health and Biosecurity, CSIRO, Canberra, Australia; 50 School of Psychological Science, University of Bristol, Bristol, UK; 51 Research Unit of Population Health, University of Oulu, Oulu, Finland; 52 Department of Medicine, Critical Care, Allergy and Sleep, University of California, PulmonarySan Francisco, San Francisco, CA USA; 53 Department of Epidemiology, Gillings School of Global Public Health, University of North Carolina at Chapel Hill, Chapel Hill, USA; 54 SAMRC Unit on Risk & Resilience in Mental Disorders, Dept of Psychiatry & Neuroscience Institute, University of Cape Town, Cape Town, South Africa; 55 Epidemiology and Population Health, Stanford School of Medicine, Stanford University, Stanford, USA; 56 Research Department of Clinical, Education and Health Psychology, Faculty of Brain Sciences, UCL, London, UK; 57 Faculty of Medicine, Nursing and Health, Psychiatry Monash Health, Monash University, Melbourne, Australia; 58 Department of Epidemiology, University Medical Center Groningen, University of Groningen, Groningen, Netherlands; 59 GRIAC Research Institute, University Medical Center Groningen, University of Groningen, Groningen, Netherlands; 60 Department of Psychology, University of Bath, Bath, UK; 61 Department of Pediatrics, Allergy, Immunology and BMT, University of California, San Francisco, San Francisco, CA USA; 62 Centre for Individualised Infection Medicine (Ciim), Helmholtz Centre for Infection Research (HZI), Hannover Medical School (MHH), Hanover, Germany; 63 Helmholtz Centre for Infection Research (HZI), TWINCORE, Hannover Medical School (MHH), Hanover, Germany; 64 Department of Biomedical Informatics, University of Colorado Anschutz Medical Campus, Aurora, USA; 65 SAMRC Unit on Child Health, Dept of Paediatrics, University of Cape Town, Cape Town, South Africa; 66 Epidemiology, Biostatistics, School of Public Health, And Environmental Health, University of Memphis, Memphis, USA; 67 Department of Social and Behavioral Science, Harvard T. H. Chan School of Public Health, Boston, USA; 68 Immunity Inflammation and Disease Laboratory, National Institute of Environmental Health Sciences, Durham, USA; 69 Generation R Study Group, Erasmus MC, University Medical Center Rotterdam, Rotterdam, Netherlands; 70 Department of Pediatrics, Erasmus MC, University Medical Center Rotterdam, Rotterdam, Netherlands; 71 Department of Epidemiology, Erasmus MC, University Medical Center Rotterdam, Rotterdam, Netherlands; 72 Department of Biomedical Data Sciences, Molecular Epidemiology, Leiden University Medical Center, Leiden, Netherlands; 73 Faculty of Psychology and Humanities, Universidad San Sebastián, Valdivia, Chile

**Keywords:** Epigenetics, Pediatrics, Child psychiatry, DNA methylation, Longitudinal analysis, Meta-analysis, ADHD, Sleep, BMI, Asthma

## Abstract

**Background:**

DNA methylation (DNAm) is a developmentally dynamic epigenetic process; yet, most epigenome-wide association studies (EWAS) have examined DNAm at only one timepoint or without systematic comparisons between timepoints. Thus, it is unclear whether DNAm alterations during certain developmental periods are more informative than others for health outcomes, how persistent epigenetic signals are across time, and whether epigenetic timing effects differ by outcome.

**Methods:**

We applied longitudinal meta-regression models to published meta-analyses from the PACE consortium that examined DNAm at two timepoints—prospectively at birth and cross-sectionally in childhood—in relation to the same child outcome (ADHD symptoms, general psychopathology, sleep duration, BMI, asthma). These models allowed systematic comparisons of effect sizes and statistical significance between timepoints. Furthermore, we tested correlations between DNAm regression coefficients to assess the consistency of epigenetic signals across time and outcomes. Finally, we performed robustness checks, estimated between-study heterogeneity, and tested pathway enrichment.

**Results:**

Our findings reveal three new insights: (i) across outcomes, DNAm effect sizes are consistently larger in childhood cross-sectional analyses compared to prospective analyses at birth; (ii) higher effect sizes do not necessarily translate into more significant findings, as associations also become noisier in childhood for most outcomes (showing larger standard errors in cross-sectional vs prospective analyses); and (iii) DNAm signals are highly time-specific, while also showing evidence of shared associations across health outcomes (ADHD symptoms, general psychopathology, and asthma). Notably, these observations could not be explained by sample size differences and only partly to differential study-heterogeneity. DNAm sites changing associations were enriched for neural pathways.

**Conclusions:**

Our results highlight developmentally-specific associations between DNAm and child health outcomes, when assessing DNAm at birth vs childhood. This implies that EWAS results from one timepoint are unlikely to generalize to another. Longitudinal studies with repeated epigenetic assessments are direly needed to shed light on the dynamic relationship between DNAm, development and health, as well as to enable the creation of more reliable and generalizable epigenetic biomarkers. More broadly, this study underscores the importance of considering the time-varying nature of DNAm in epigenetic research and supports the potential existence of epigenetic “timing effects” on child health.

**Supplementary Information:**

The online version contains supplementary material available at 10.1186/s13073-025-01451-7.

## Background

DNA methylation (DNAm) is an important epigenetic regulator of development and health. DNAm is influenced by genetic [1, 2] and environmental factors, beginning in utero (e.g., maternal smoking [3], stressful life events [4], air pollution [5], or physical activity [6]). DNAm alterations have also been linked to a wide range of health outcomes across childhood, including asthma [7], attention-deficit/hyperactivity disorder (ADHD) symptoms [8], and body mass index (BMI) [9]. Together, these properties make DNAm an attractive biological process in the search for biomarkers and mediators of disease risk.

DNAm is highly dynamic during development making it particularly interesting, but also challenging to study. Over half of DNAm sites show changes in methylation from birth to 18 years of age [10]. Furthermore, in around a third of DNAm sites, the degree of change varies between individuals, perhaps reflecting exposure to different postnatal environments, genetic variation, or stochastic processes [11]. Yet, most studies linking DNAm to health phenotypes measure DNAm only once [12]. Thus, it is largely unknown (i) whether the relationship between DNAm and health outcomes varies across development (ii) at which developmental periods DNAm profiles could be most informative for these outcomes, and (iii) to what extent DNAm-health associations at one timepoint can be generalized to other timepoints.

In most pediatric population studies, DNAm is either measured in cord blood samples at birth and associated with a child outcome at a later timepoint (i.e., prospective epigenome-wide association study [EWAS]) *or* DNAm is measured from a blood sample at the same timepoint as the child outcome (i.e., cross-sectional EWAS). Theoretical arguments exist for either design. On the one hand, DNAm measured at birth coincides with a developmentally sensitive period and may reflect causal effects of genetic and in utero environmental factors influencing the risk of later outcomes [13]. Furthermore, reverse causation scenarios are less likely, given that outcomes in childhood are unlikely to affect methylation profiles at birth. However, cross-sectional EWASs during childhood may result in a stronger association signal, due to the temporal proximity between predictor and outcome, a larger accumulation of environmental effects (prenatal and postnatal), or the potential for DNAm patterns to reflect both causes and consequences of health (reverse causality). Cord and peripheral blood also represent different tissues, with different cell compositions (e.g., nucleated red blood cells being present in cord blood), which may contribute to association differences [14, 15]. However, as cord blood is only available at birth, and early cell-type changes are in part developmentally regulated, separating the influence of tissue versus timing is challenging [15, 16].

Recently, the Pregnancy And Childhood Epigenetics (PACE) Consortium [17] published five multi-cohort EWAS meta-analyses that investigated DNAm using *both designs* in relation to the same child outcome, spanning mental and physical health domains, namely: ADHD symptoms [8], general psychopathology (measured as a latent factor; GPF) [18], sleep duration [19], BMI [9], and asthma [7]. Results from these previous studies can be summarized as follows (Table 1): for ADHD symptoms, there were more hits for DNAm at birth rather than in childhood (i.e., prospective EWAS showed more hits than cross-sectional EWAS); whereas the opposite was true for BMI and asthma (i.e., prospective EWAS showed fewer hits than cross-sectional EWAS). For GPF and sleep duration, results were mostly null at either timepoint. Together, these findings point to the potential existence of epigenetic “timing effects” on child health.

**Table 1:** Published epigenome-wide association studies of child developmental outcomes from PACE, jointly re-analyzed in the present study

Study	Outcome	Age	Instrument	Methylation	Model	Covariates	Meta-analysis	Sig. threshold	Birth EWAS		Childhood EWAS	
									*n*	*n*_cpg_	*n*	*n*_cpg_
Neumann et al. (2020) [8]	ADHD	5–15	Paternal questionnaire	Beta	LMM, LM	Sex, gest. age, age^a^, mat. age, mat edu., mat. smoking, cell prop., batch	HE-RE	Bonf	2477	9	2374	0
Rijlaarsdam et al. (2023) [18]	GPF	6–12	Paternal questionnaire	Beta (Trimmed)	RLM	Sex, gest. age, age, mat. age, mat. edu., mat. smoking, cell prop., ancestry, batch	FE	Bonf	2178	0	2190	1
Sammallahti et al. (2022) [19]	Sleep	4–13	Paternal questionnaire	Beta (Trimmed)	LM	Sex, age, mat. age, mat. edu., mat. smoking, cell prop., ancestry, batch	FE	FDR	3658	0	2539	0
Vehmeijer et al. (2020) [9]	BMI	2–10	Measured	Beta	RLM	Sex^b^, gest age, age^b^, mat. age, mat edu., mat. smoking, mat. BMI, parity, birth weight^a^, breastfeeding^a^, cell prop., ancestry, batch	FE	Bonf. (FDR)	4641	1 (1)	3406	1 (10)
Reese et al. (2019) [7]	Asthma	5–17	Diagnosis	Beta	Logistic	Sex, mat. age, mat. edu., mat. smoking, cell prop., batch	FE	FDR	3572	9	2834	179

^a^Covariate only used in school-age analyses

^b^Covariates considered in the creation of BMI standard deviation scores

*ADHD *attention deficit hyperactivity disorder, *GPF *general psychopathology factor, *BMI *body mass index, *Age *age at outcome, *LMM *linear mixed model, *LM *linear model (OLS), *RLM *robust linear model, *Logistic *logistic regression model, *mat*. maternal, *edu*. education, *prop*. proportion, *HE-R*E Han & Eskin Random Effects Model, *FE *fixed effects model, *n*_*cpg*_ number of CpG sites genome-wide significant, *n *sample size

Despite these intriguing findings, the studies’ main goal was to maximize the identification of health-relevant DNAm sites at each timepoint, rather than systematically quantify temporal changes of DNAm-health associations. Addressing this aim requires analyses that were not originally performed, including quantitatively comparing effect sizes between timepoints, accounting for sample size imbalances that affect statistical power per timepoint, and examining statistical and biological factors contributing to temporal differences in DNAm-health associations. Furthermore, no comparison has been performed *across* studies, to establish how temporal patterns may vary for different health outcomes, and whether methylation signals for one outcome correlate with that for other outcomes (indicating pleiotropy/shared epigenetic effects).

Here, we re-analyzed the five PACE meta-analyses on ADHD symptoms, GPF, sleep duration, BMI, and asthma to explore timing effects on DNAm-health associations during development. For each outcome, we integrated results from the prospective EWAS (cord blood DNAm at birth) and the cross-sectional EWAS (whole blood DNAm in childhood) into a longitudinal meta-regression model. This model systematically quantified changes in effect sizes and statistical significance between timepoints, and we also explored a range of factors that may contribute to the observed temporal trends. We then correlated DNAm associations *between timepoints* (to assess the generalizability of epigenetic signals from one timepoint to another) and *across health outcomes* (to explore the presence of shared DNAm associations).

## Methods

### Participating cohorts

We requested cohort-level epigenome-wide summary statistics from five meta-analytic studies previously performed by the PACE Consortium. We obtained permission for re-analysis from the meta-analysis leads and representatives of all originally participating cohorts, except for the GOYA study, which was excluded here from further analysis. Cohort-level summary statistics were obtained from the meta-analysis leads through personal correspondence. Respective local ethics committees previously approved the included studies [7–9, 18, 19].

In total, we included 26 cohorts with pooled sample sizes ranging from 2178 to 4102 participants per outcome. Additional file 1: Tables S1 and S2 show an overview of included cohorts and the overlap between timepoints/outcomes, see also original publications for details [7–9, 18, 19]. All cohorts were population-based studies with no inclusion/exclusion criteria based on diagnostic status or medication use.

### Data

EWAS summary statistics included the association between DNAm (predictor) and four different continuous outcomes (ADHD symptoms, GPF, sleep duration, and BMI) and one categorical outcome (asthma diagnosis). Regression statistics were available for both prospective analyses and cross-sectional analyses. Prospective here refers to associating DNAm at birth with the phenotype in childhood, whereas cross-sectional refers to associating DNAm measured at the same age as a continuous outcome, or in case of asthma, symptoms or medication use up to 1 year prior. See Table 1 and original publications [7–9, 18, 19] for age distributions per outcome.

DNAm was either measured in cord blood at birth or in peripheral blood in childhood with either Illumina 450 K or EPIC arrays (although only 450 K DNAm sites remained after QC, see below). Predictors were the DNAm betas ranging from 0 to 1, corresponding to 0 to 100% methylation. In the case of GPF and sleep duration, the studies trimmed DNAm outliers outside the range of [25th percentile − (3*interquartile range (IQR) to 75th percentile + 3*IQR) based on the analytical choices made in the previously published work.

ADHD symptoms and GPF were assessed via parental questionnaires. For both outcomes, most participants were scored by either the Child Behavior Checklist (CBCL) or the Development and Well-being Assessment (DAWBA). For ADHD symptoms, DSM-oriented ADHD scales were used [8]. The GPF, on the other hand, was a latent variable inferred from all internalizing, externalizing, thought, and other symptom subscales featured on the instruments [18]. Sleep duration was determined by parental reports on either hours slept or calculated from reported falling asleep and wake-up times [19]. BMI was computed based on measured height and weight [9]. These four continuous outcomes were *z*-score standardized within each cohort to account for questionnaire differences and other study-specific effects [8, 9, 18, 19]. Asthma was analyzed dichotomously with asthma status based on a doctor’s diagnosis. Symptoms or medication use had to be present currently or in the year prior to assessment [7]. All EWAS were adjusted for sex, maternal age, maternal education, maternal smoking, cell proportions, and possible batch effects, in addition to other variables, which differed depending on outcome and time-point (Table 1). A variety of analysis models were employed, such as ordinary-least square (OLS) linear models (sleep duration), robust linear models (GPF, BMI), and logistic regression (asthma) (Table 1). For ADHD symptoms, two cohorts used OLS regression and otherwise linear mixed models with a random effect for batch. In the case of prospective analyses, multiple childhood ADHD measures were available for three cohorts. These cohorts added a participant-level random effect to account for repeated measures. For cross-sectional analyses, a single ADHD assessment at the same age as DNAm measurement was chosen [8].

Each cohort had performed conventional quality control, such as detection thresholds, removal of probes with failed bisulfate conversion, hybridization, and extension; sex checks; and call rate filters; see the original publications for details [7–9, 18, 19]. We applied the following additional quality control: (1) kept only autosomal DNAm sites, (2) removed DNAm sites with information in less than three cohorts or 1000 participants per time-point, (3) kept only CpG sites present both at birth and in childhood, (4) removed cross-reactive probes using the maxprobe 0.0.2 package [20]. Finally, to examine whether the differences in statistical significance were influenced by sample size differences, we also performed sensitivity analyses with similar sample sizes at both timepoints. We removed (combination of) cohorts which resulted in the most similar sample sizes between cohorts (Additional file 1: Table S1).

### Statistical analysis

Each summary statistic contained information on the regression coefficient (*β*_jk_) and SE. *β* represents the difference in child health outcomes in standard deviations (SD) between no to full methylation in the case of continuous variables or in odds ratio for the categorical outcome asthma. *β* is given per DNAm assessment timepoint *j* (birth or childhood) estimated in cohort *k*. We applied multi-level meta-regressions to pool effect sizes across cohorts and to model changes in effect sizes depending on DNAm assessment time-point. This model therefore quantified the DNAm associations at birth, in childhood, as well as the differences in associations between timepoints. Repeated measures from cohorts that contributed association estimates for both DNAm at birth and in childhood were taken into account with a random intercept. The main model took the form of:

$$\beta_{jk}\;=\;\beta_{\mathrm{birth}}\;+\;\beta_{\triangle\mathrm{childhood}}\;+\;u_{k\;}+\;r_k$$

*β*_birth_ is the intercept and represents the pooled variance-weighted associations of methylation at a CpG site on an outcome at birth or childhood, respectively.

*β*_Δchildhood_ refers to the change in association from DNAm at birth to childhood.

*u*_*k*_ is the study random effect and refers to the deviation of the mean associations within cohort k from overall mean associations.

*r*_*k*_ denotes a residual error.

We also ran a statistically identical model with reverse time direction to extract DNAm effects in childhood. We applied these meta-regression models to each DNAm site separately using metafor 4.2.0 [21] in R 4.2.2 [22]. After estimating the associations and their change for each CpG site, we aggregated statistics across the genome to characterize global trends. Specifically, we examined across all CpG sites the mean absolute effect size at birth $\left(\left|{\overline\beta}_{\text{birth}}\right|\right)$, mean absolute effect size in childhood $\left(\left|{\overline\beta}_{\text{childhood}}\right|\right)$, and the mean effect size difference between birth and childhood $\left(\left|{\overline\beta}_{\triangle {\text{childhood}}}\right|\right)$. In addition, we examined trends of statistical significance by taking the mean *z* test statistic of *β*_birth_ $\left(\left|{\overline z}_{\text{birth}}\right|\right)$ and *β*_childhood_ $\left(\left|{\overline z}_{\text{childhood}}\right|\right)$, representing the evidence of association for DNAm at birth and childhood, respectively. Furthermore, we also characterized the change in mean statistical significance from birth to childhood methylation $\left(\triangle\overline z\right)$. The use of absolute values makes it possible to aggregate effect size magnitudes across different effect direction patterns, but such statistics by design do not distinguish between directions. We therefore also classified all DNAm sites showing a nominally significant change into nine different effect direction categories: Pos/Pos, Pos/Null, Null/Pos, Neg/Neg, Neg/Null, Null/Neg, Pos/Neg, Neg/Pos, and Null/Null. Pos and Neg here refer to a positive or negative association above the 80% quantile at birth/childhood; otherwise, they are referred to as null.

We also examined whether between-study heterogeneity changed between birth and childhood estimates by adding a random slope of *β*_Δchildhood_ on the cohort level. We extracted *τ*, which indicates to which degree DNAm effects vary due to between-study heterogeneity within 1SD. In other words, assuming no sampling error and normal distribution, 67% of estimates are expected to be within *β* + -*τ* due to study differences. Reported correlations are Spearman correlations. GO term enrichment for DNAm sites with nominally significant change and nominally significant association for at least one timepoint was tested using missMethyl 1.36.0. [23, 24].

## Results

### Quantifying change in EWAS effect sizes from birth to childhood

**Fig. 1:**
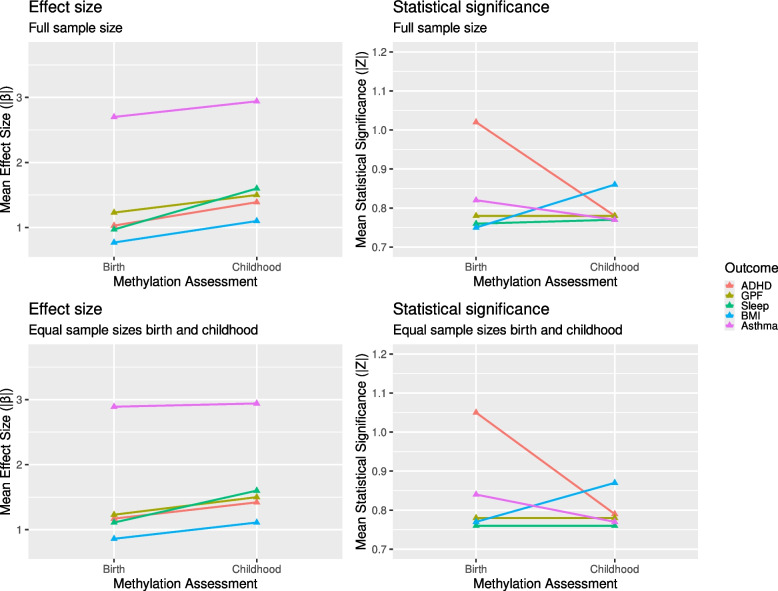
Mean effect sizes and statistical significance for DNAm at birth and in childhood. Mean effect sizes (left column) and mean statistical significance (right column) across all tested autosomal DNAm sites per outcome (color) and timepoint. Upper row displays results from analyses utilizing the maximum available sample sizes. Lower row displays results from analyses with cohorts removed to achieve equal sample sizes at both timepoints. Effect size is given as absolute regression coefficient (|‾*β*|), representing the difference in child health outcomes in SD between full or no methylation in the case of continuous outcomes (ADHD, general psychopathology, sleep duration, and BMI), or log(odds ratio) for categorical outcomes (asthma diagnosis). Statistical significance is given as mean absolute *Z*-values

**Fig. 2:**
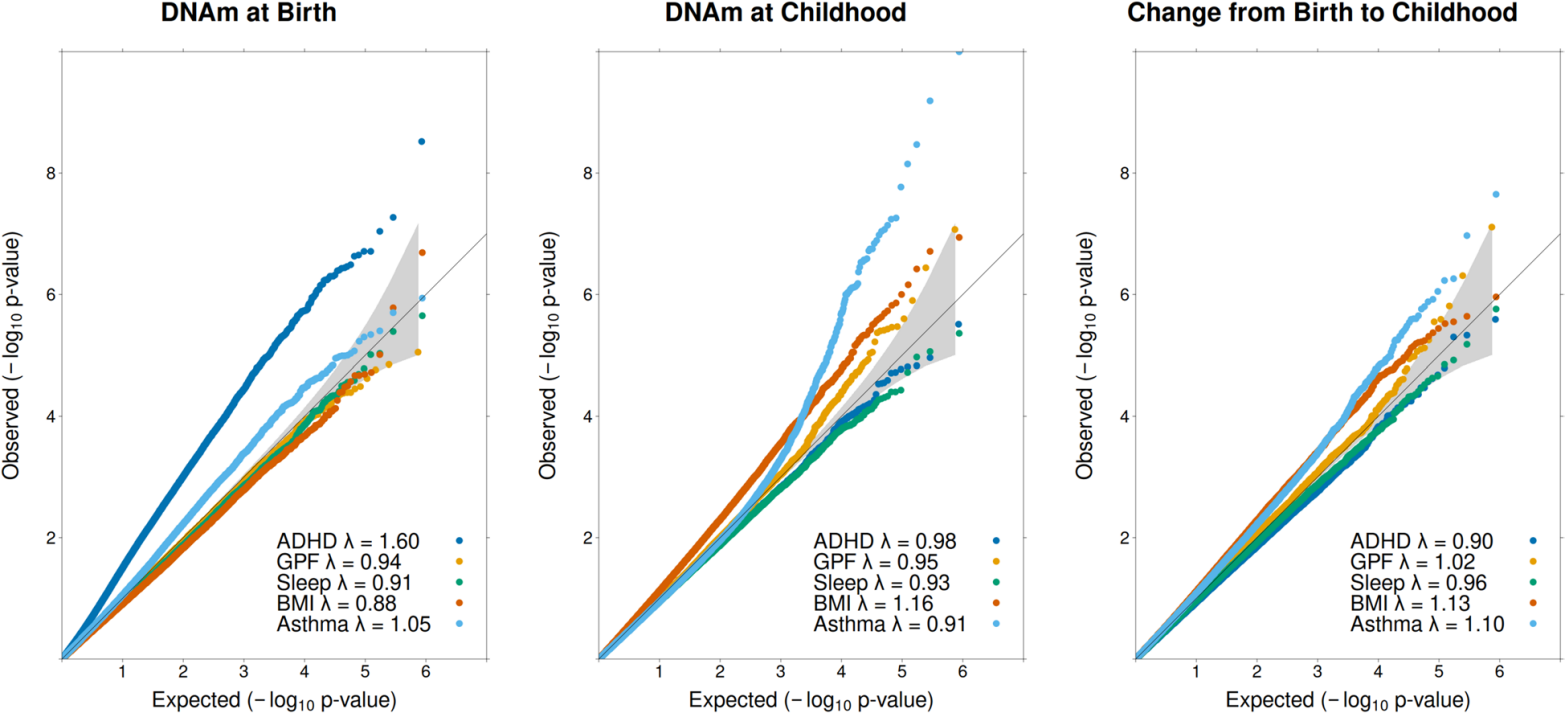
QQ-plots. Distribution of observed *p*-values (*y*-axis) vs expected (*x*-axis). Diagonal represents the expected distribution of *p*-values by chance. Upwards deviations indicate a higher presence of lower *p*-values than expected assuming a null effect. Distributions are given for DNAm effects at birth (left), in childhood (middle), and for change in effect between birth and childhood (right) per outcome (color). Gray displays the 95% confidence interval of the null distribution

For DNAm at birth, mean effect sizes across DNAm sites ranged from 0.77SD (BMI) to 1.23SD (GPF) for continuous measures (Table 2; Figs. 1 and 2; Additional file 2: Fig. S1, S2). Averaged across phenotypes, 10% higher methylation was associated with a 0.10SD outcome difference. For asthma, mean log(odds) were 2.70, which corresponds to a 10% methylation difference being associated with 1.30 lower/higher odds of receiving an asthma diagnosis.

**Table 2:** Association between DNA methylation either at birth or in childhood and child developmental outcomes (full sample)

Outcome	*n*_cpg_	DNAm at birth (prospective EWAS)						DNAm in childhood (cross-sectional EWAS)						Change between time points				
		*n*	*n*_cohorts_	Mean *β* (abs.)	Mean SE	Mean *z*	*n*_cpg_ *p* < 0.05 (FDR/bonf.)	*n*	*n*_cohorts_	Mean *β* (abs.)	Mean SE	Mean *z*	*n*_cpg_ *p* < 0.05 (FDR/bonf.)	*n*_cohorts_ Both	Δ*β*	*N*_cpg_ + Δβ	*N*_cpg_ − Δβ	Δ*z*
ADHD	430,327	2477	6	1.03	1.10	1.02	57,339 (896/3)	2374	5	1.39	1.76	0.78	19,034 (0/0)	3	0.36	10,542 (0/0)	6841 (0/0)	− 0.23
GPF	372,278	2178	4	1.23	1.59	0.78	16,549 (0/0)	2190	5	1.50	1.98	0.78	17,767 (1/1)	3	0.27	13,375 (1/1)	6475 (0/0)	0.01
Sleep	431,159	3658	10	0.97	1.30	0.76	17,399 (0/0)	2539	5	1.60	2.06	0.77	18,113 (0/0)	4	0.63	14,447 (0/0)	5171 (0/0)	0.01
BMI	435,652	4102	14	0.77	1.04	0.75	16,012 (0/0)	3406	11	1.10	1.29	0.86	30,615 (2/1)	6	0.33	23,493 (0/0)	6634 (0/0)	0.11
Asthma	432,728	3065 (631)	7	2.70	3.44	0.82	26,112 (0/0)	2834 (631)	9	2.94	3.92	0.77	18,605 (66/11)	0	0.24	18,024 (2/2)	9576 (0/0)	− 0.06

*ADHD* attention deficit hyperactivity disorder, *GPF* general psychopathology factor, *BMI* body mass index, *n*_*cpg*_ number of CpG sites tested, *n* sample size (cases), *n*_*cohorts*_ number of cohorts, *mean β (abs.)* the mean absolute regression coefficient across DNAm sites (*β* represents the expected difference in the outcome in SD when CpG sites are fully methylated compared to no methylation. For asthma, *β* represents the log(odds) difference), *Mean SE* mean standard error, *Mean z* mean *z* values across CpG sites. *z* = β/SE indicating statistical significance, *n*_*cpg*_*p<0.05 (FDR/bonf.)* number of nominally significant CpG sites (after adjustment for false discovery rate/after Bonferroni adjustment), *n*_*cohorts*_*both* number of cohorts which contributed to both birth and school age analyses, *Δβ* change in effect size from birth to school age, *N*_*cpg*_*+Δβ*number of CpG sites with increasing effect size and nominally significant change (FDR/Bonferroni), *N*_*cpg*_*−Δβ* number of CpG sites with decreasing effect size and nominally significant change (FDR/Bonferroni)

Compared to DNAm at birth, mean effect sizes for DNAm in childhood were consistently *higher* across all tested outcomes (Tables 2 and 3; Figs. 1 and 2; Additional file 2: Fig. S1, S2), ranging from 1.10SD (BMI) to 1.60SD (sleep duration) for continuous outcomes and a log(odds) of 2.94 (odds ratio of 1.34) for asthma. When quantifying this *difference* in effect sizes between birth and childhood, the smallest mean difference was observed for BMI $\left(\left|{\overline\beta}_{\text{birth}}\right|=0.77\ {\text{vs}}\ \left|{\overline\beta}_{\text{childhood}}\right|=1.10\right)$ and the largest difference for sleep duration $\left(\left|{\overline\beta}_{\text{birth}}\right|=0.97\ {\text{vs}}\ \left|{\overline\beta}_{\text{childhood}}\right|=1.60\right)$. Aggregating across continuous outcomes, mean effect sizes were 40% higher in childhood, resulting in a mean outcome difference of 0.14SD per 10% methylation. Additional file 1: Table S3 shows effect size comparisons across percentiles.

**Table 3:** Comparison of birth EWAS (i.e., prospective analysis) versus childhood EWAS (i.e., cross-sectional analysis): Overview of study findings

Outcome	Change from birth to childhood EWAS		Potential contributing factors		Sensitivity analyses		Correlation analyses	
	Effect size	Statistical significance	EWAS sample size	Between-study heterogeneity	Do results hold when making N equal across time points?	Do results hold within single longitudinal cohort (ALSPAC)	Correlations between time points (i.e., stability)	Correlations with other phenotypes
ADHD	↑	↑	=	↑	✓	✓	✓ (*r*_*s*_ = 0.31)	✓ (*r*_*s*_ = − 0.18–0.35)
GPF	↑	=	=	=	✓	✓	≠ (*r*_*s*_ = 0.08)	✓ (*r*_*s*_ = − 0.07–0.35)
Sleep	↑	=	↓	↑	✓	✓	≠ (*r*_*s*_ = 0.06)	≠ (*r*_*s*_ = − 0.04–0.06)
BMI	↑	↑	↓	↑	✓	✓	≠ (*r*_*s*_ = 0.05)	✓ (*r*_*s*_ = − 0.18–0.06)
Asthma	↑	↑	=	↑	✓	✓	≠ (*r*_*s*_ = − 0.04)	✓ (*r*_*s*_ = − 0.16–0.21)

While these effect size figures provide a global view of genome-wide association change, they do not take into account statistical precision (standard error (SE)). Another way to quantify DNAm differences at birth versus in childhood is by counting the number of sites at which DNAm effect sizes increase or decrease over time based on a *p*-value threshold of change. Among probes that showed at least a nominally significant difference between timepoints, there were 1.5–3 × more DNAm sites with a larger as opposed to smaller effect size in childhood across health outcomes (Table 2, Fig. 2, Additional file 2: Fig. S1–S4). To test the robustness of this approach, we also examined the ratio of DNAm sites that show an effect size increase vs decreases over time across different change *p*-value thresholds from no thresholding to *p* < 0.0001 (Additional file 2: Fig. S4). We observed that the ratio is always positive (more DNAm showing an increase in effect size over time)—a trend that becomes stronger as the threshold becomes more stringent (lower *p*-values).

For the DNAm sites that showed nominally significant change over time, we also examined the direction of association with health outcomes, and whether this direction was consistent across timepoints. The most common pattern was a null or small effect at birth, followed by a positive association in childhood (Additional file 1: Table S3). This applied to all outcomes, except BMI. Here the most frequent pattern was a switch from a positive association at birth to a negative association in childhood.

Three DNAm sites showed a genome-wide significant change in association. Cg11945228 in *BRD2* had no evidence for association with GPF at birth (*β*_birth_ = 5.28, SE = 3.76, *p* = 0.16), but was associated in childhood (*β*_childhood_ = − 37.00, SE = 6.91, *p* = 8.58*10^−8^), a significant change (*p* = 7.68*10^−8^). Similarly, cg10644885 in *ACP5* had a significant change (*p* = 2.25*10^−8^) from no association with asthma at birth (*β*_birth_ = − 0.56, SE = 1.19, *p* = 0.64) to significance in childhood (*β*_childhood_= − 15.00, SE = 2.29, *p* = 5.57*10^−11^). In addition, cg22708087 in *FRY* changed from a positive association with asthma at birth (*β*_birth_ = 7.47, SE = 1.80, *p* = 3.42*10^−5^) to a negative association in childhood (*β*_childhood_ = − 12.64, SE = 3.32, *p* = 1.44*10^−4^). This change was genome-wide significant (*p* = 1.06*10^−7^). For all three DNAm sites, absolute effect sizes were larger in childhood.

### Testing the relationship between effect size and the ability to identify significant associations

While mean effect sizes were robustly larger for DNAm in childhood compared to DNAm at birth for all outcomes, this did not necessarily translate into more significant associations, as quantified by higher *z* test-statistics (lower *p*-values) (Tables 2 and 3; Figs. 1 and 2; Additional file 2: Fig. S1, S2).

#### ADHD symptoms

DNAm at birth showed the strongest association signal with ADHD symptoms, as evidenced by a mean *z*-value of 1.02 and the identification of the largest number of significant associations at all tested thresholds (Bonferroni/FDR/nominal). Despite an increase in effect sizes from birth to childhood, the mean z-value dropped (1.02 at birth vs 0.78 in childhood). Three DNAm sites were significant after Bonferroni correction at birth, but no CpG site was identified as genome-wide significant in childhood neither with FDR nor Bonferroni correction. Furthermore, the number of nominally significant sites was threefold lower in childhood (*n*_cpg-birth_ = 57,339 vs *n*_cpg-childhood_ = 19,034). Among all outcomes, ADHD showed the highest lambda for birth DNAm (*λ* = 1.60), which can either indicate a high polygenic signal in a well-powered sample, unmeasured confounding, or both. We had previously investigated this issue [8] and concluded that the inflation stems most likely from a true signal based on the following observations: (1) reducing sample size by 1/3 effectively eliminated inflation, (2) BACON [25] analyses suggested that inflation of the *p*-value distribution can be mostly attributed to a true signal rather than spurious inflation, (3) systematic genome-wide confounding biases would most likely affect birth and school-age methylation similarly, but inflation is only seen for birth DNAm.

#### GPF

The mean z-value remained constant at 0.78 for both timepoints, and the number of nominally significant sites remained similar. No DNAm site reached genome-wide significance at birth, and one DNAm site reached genome-wide significance when assessed in childhood.

#### Sleep duration

Mean *z*-values for sleep duration did not differ between timepoints (0.76 at birth and 0.77 at school-age) and the number of nominally significant sites remained similar, with no genome-wide significant findings at either timepoint.

#### BMI

For BMI the higher DNAm effect sizes in childhood corresponded with a higher statistical significance, with mean z-values increasing from 0.75 at birth to 0.86 in childhood. This is also reflected by the doubling of nominally significant associations from birth to childhood (16,012 to 30,615), as well as by the presence of one genome-wide hit in childhood (Bonferroni correction), but no genome-wide significant DNAm sites at birth.

#### Asthma

The mean *z*-values and number of nominally significant sites were somewhat larger at birth (*z* = 0.82) than in childhood (*z* = 0.77). While this reflects the genome-wide trend, it is important to emphasize that the number of probes with genome-wide significance was much larger for DNAm in childhood (0 hits at birth vs 11 in childhood, after Bonferroni correction).

#### What explains these outcome-specific patterns?

We searched for potential explanations for why statistical significance did not necessarily increase over time, or even decreased, despite effect size increases. *Z*- and *p*-values represent the ratio between effect size and statistical uncertainty. We found that SE increased from birth to childhood either to a disproportionately larger (ADHD symptoms, asthma) or similar (GPF, sleep duration) extent as the effect size increased (Tables 2 and 3), i.e., only for BMI did the increase in effect size outpace the increase in SE leading on average to more statistical significance.

Next, we investigated potential sources for the SE increase. The first was *sample size*, which was unequal between timepoints for some outcomes. For GPF, the total sample size was very similar, and for asthma, the number of cases was equal between timepoints. However, especially for sleep duration and BMI, sample sizes were much lower for DNAm measured in childhood, which increases SE. In sensitivity analyses, we removed cohorts (Additional file 1: Table S1) to achieve equal sample sizes between timepoints. Interestingly, patterns remained largely the same, i.e., with only BMI showing corresponding increases in both effect sizes and statistical significance over time (Additional file 1: Table S4).

Second, we examined *between-study heterogeneity*, which tends to increase SE. We fit random slope models, allowing for different amounts of heterogeneity at different DNAm assessment periods. Between-study heterogeneity increased for all outcomes over time, except for GPF (Additional file 1: Table S5), suggesting that it may partly influence differences in EWAS signal between timepoints. At the same time, re-computing meta-regression analyses using a single cohort (ALSPAC, the largest cohort contributing to all analyses with similar sample sizes at birth and childhood) led to similar results as the meta-analysis (Additional file 1: Table S6), suggesting that observed temporal differences are unlikely to be solely explained by cohort composition in the meta-analyses.

### Estimating correlations in epigenetic signals across timepoints and child outcomes

**Fig. 3:**
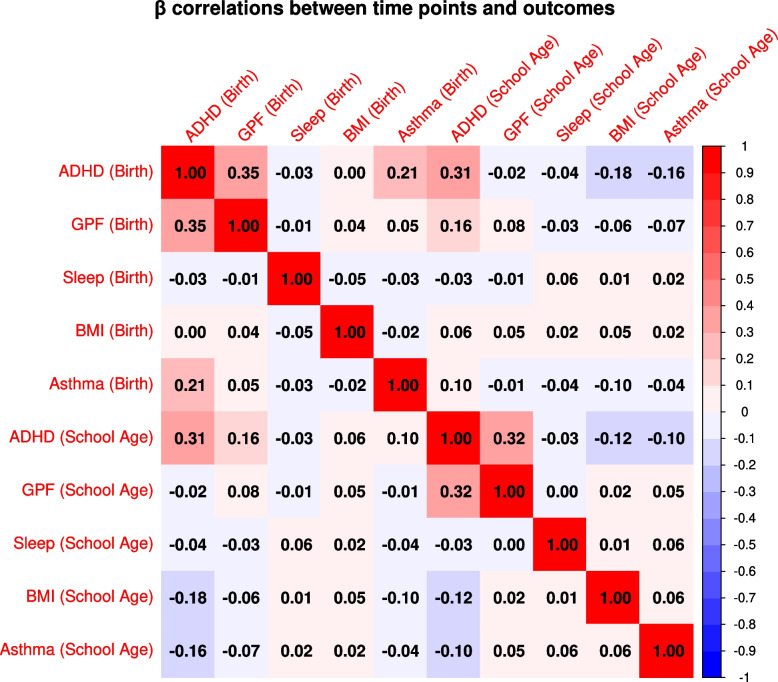
Correlations between DNAm effects at birth and childhood and across outcomes. This correlation matrix displays Spearman correlations between regression coefficients for DNAm at birth and childhood and across outcomes. Intensity of red represents higher positive correlations and blue lower negative correlations

To test the consistency of epigenetic associations over time and across outcomes, we computed Spearman correlations (*r*s) between the regression coefficients of all timepoints and outcomes (Fig. 3). For ADHD symptoms, estimates at birth correlated modestly with those in childhood (*r*s = 0.31). For all other outcomes, estimates between timepoints were uncorrelated (*r*s < 0.08). The coefficients in the ADHD symptoms analysis correlated most with the coefficients for other outcomes. For instance, the EWAS signal at birth for ADHD symptoms was positively correlated with the signal at birth for GPF (*r*s = 0.35) and asthma (*r*s = 0.21), but negatively correlated with the EWAS signal in childhood of BMI (*r*s = − 0.18) and asthma *(rs* = − 0.16).

Overlap between cohorts contributing to analyses at the same timepoint tended to be larger than between timepoints (Additional file 1: Table S2). This may have led to an underestimation of correlations between timepoints. To test this, we re-ran correlation analyses within ALSPAC and found that between timepoint correlations remained low for GPF, sleep duration, and BMI (*r*s < 0.12) and modest for ADHD symptoms (*r*s = 0.25) (Additional file 2: Fig. S5). Asthma could not be tested, due to unavailable analyses in childhood.

### Pathway enrichment analyses for health-related DNAm patterns showing change from birth to childhood

We performed gene ontology enrichment analyses to probe the potential biological relevance of temporal changes in DNAm-health associations. A secondary aim was to examine the possibility that we may be mainly picking up tissue differences (as opposed to developmental/temporal differences) between birth and childhood assessments (i.e., cord vs peripheral blood). For example, if we were to identify enrichment of blood or cell-type specific terms (e.g., leukocyte differentiation), this could point at cell-type composition differences between cord and peripheral blood primarily driving observed changes in DNAm associations. On the other hand, enrichment for more outcome-specific pathways (e.g., neuron differentiation) may instead indirectly lend further support for the involvement of developmental processes, independent of blood tissue differences. We selected sites that (i) were nominally associated with an outcome at either timepoint *and* (ii) showed at least nominally significant *change* in associations from birth to childhood. Notably for ADHD symptoms, GPF, and sleep duration, neural features stand out among the top 10 pathways (e.g., cerebral cortex and neuron development, synapses, and dendrites; Table 4). While neural pathways also rank highly for BMI and asthma, other more general cell processes such as morphogenesis are prominently represented. However, no pathway was significant after adjustment for multiple testing of all 22,560 GO terms. See additional file 1: Tables S6–S11 for all pathways with nominal significance.

**Table 4:** Gene ontology enrichment analyses: top 10 terms for phenotype-associated DNAm sites showing change from birth to childhood

	ADHD		GPF		Sleep		BMI		Asthma	
*n*_cpg_	10,742		11,228		11,591		19,055		18,771	
Ontology	Term	*p*-value	Term	*p*-value	Term	*p*-value	Term	*p*-value	Term	*p*-value
**Biological processes (BP)**										
BP	Vesicle cytoskeletal trafficking	1.0E − 03	Neurogenesis	2.2E − 04	Proximal/distal pattern formation	3.6E − 04	Positive regulation of RNA biosynthetic process	4.5E − 05	Cell morphogenesis	3.6E − 05
BP	G protein-coupled glutamate receptor signaling pathway	2.7E − 03	Generation of neurons	1.0E − 03	Cell morphogenesis involved in neuron differentiation	5.8E − 04	Morphogenesis of an epithelium	4.8E − 05	Cell junction organization	1.4E − 04
BP	Cerebral cortex development	3.3E − 03	Neuron projection development	1.6E − 03	Anatomical structure arrangement	1.7E − 03	Nervous system development	4.8E − 05	Plasma membrane-bounded cell projection morphogenesis	2.1E − 04
BP	Toll-like receptor 2 signaling pathway	4.3E − 03	Platelet-derived growth factor receptor signaling pathway	1.6E − 03	Cranial nerve morphogenesis	1.7E − 03	Positive regulation of DNA-templated transcription	4.9E − 05	Cell projection morphogenesis	2.6E − 04
BP	Mucus secretion	4.7E − 03	Neuron differentiation	1.7E − 03	Central nervous system development	1.8E − 03	Regulation of cell projection organization	9.0E − 05	Cellular localization	3.6E − 04
BP	Peptidyl-threonine dephosphorylation	6.5E − 03	Neuron development	1.9E − 03	Brain development	2.1E − 03	Dendritic spine morphogenesis	1.1E − 04	Neuron projection morphogenesis	3.7E − 04
BP	Response to nutrient	6.7E − 03	Regulation of endoplasmic reticulum stress-induced intrinsic apoptotic signaling pathway	1.9E − 03	Regulation of DNA-templated DNA replication	2.5E − 03	Regulation of plasma membrane-bounded cell projection organization	1.1E − 04	Cell junction assembly	6.6E − 04
BP	Corticosteroid hormone secretion	7.0E − 03	Regulation of Notch signaling pathway	2.2E − 03	Chorionic trophoblast cell differentiation	3.0E − 03	Cell morphogenesis involved in neuron differentiation	1.1E − 04	mRNA export from nucleus	7.0E − 04
BP	Regulation of lysosome size	7.1E − 03	Negative regulation of T cell-mediated immunity	2.2E − 03	Head development	3.3E − 03	Cell morphogenesis	1.4E − 04	Adherens junction organization	8.0E − 04
BP	Modification of postsynaptic actin cytoskeleton	7.8E − 03	Negative regulation of DNA-templated transcription initiation	2.3E − 03	Skeletal system morphogenesis	3.4E − 03	Neuron differentiation	1.4E − 04	Cell part morphogenesis	8.1E − 04
**Cellular components (CC)**										
CC	Synapse	1.0E − 03	Cell junction	6.7E − 03	Dendritic tree	9.9E − 06	Cell-substrate junction	1.4E − 05	Nucleoplasm	2.3E − 04
CC	Neuron spine	1.5E − 03	Neurofilament	7.5E − 03	Dendrite	1.6E − 05	Focal adhesion	2.3E − 05	Exon-exon junction complex	1.3E − 03
CC	Dendritic spine	1.6E − 03	Protein phosphatase type 2A complex	9.0E − 03	Dendritic spine	2.8E − 05	Glutamatergic synapse	3.5E − 05	Nuclear body	2.3E − 03
CC	Postsynapse	2.3E − 03	Cell leading edge	1.0E − 02	Neuron spine	5.2E − 05	Anchoring junction	5.0E − 04	Adherens junction	2.8E − 03
CC	Plasma membrane protein complex	3.4E − 03	Synaptic membrane	1.0E − 02	Somatodendritic compartment	6.7E − 04	Cell leading edge	7.7E − 04	Sarcoplasm	2.9E − 03
CC	Voltage-gated potassium channel complex	4.2E − 03	Glutamatergic synapse	1.1E − 02	Neuron-to-neuron synapse	1.7E − 03	Actin-based cell projection	1.2E − 03	Cell leading edge	3.0E − 03
CC	Cell junction	4.4E − 03	Leading edge membrane	1.2E − 02	Asymmetric synapse	2.4E − 03	Postsynapse	1.3E − 03	Junctional sarcoplasmic reticulum membrane	4.3E − 03
CC	Potassium channel complex	5.0E − 03	Cell tip	1.3E − 02	Postsynaptic density	2.4E − 03	Cell cortex	2.7E − 03	Chromosomal region	5.4E − 03
CC	BORC complex	7.1E − 03	Postsynaptic cytoskeleton	1.4E − 02	Neuron projection	2.5E − 03	Adherens junction	3.1E − 03	Cell junction	6.5E − 03
CC	Axolemma	7.7E − 03	Eukaryotic translation initiation factor 3 complex, eIF3m	1.5E − 02	Main axon	6.3E − 03	Lamellipodium	3.2E − 03	Postsynapse	6.9E − 03
**Molecular functions (MF)**										
MF	Phospholipase binding	1.7E − 03	Lysine-acetylated histone binding	4.7E − 04	Ubiquitin-conjugating enzyme binding	2.4E − 03	Transcription factor binding	2.4E − 04	Protein serine kinase activity	9.2E − 04
MF	Efflux transmembrane transporter activity	3.7E − 03	Acetylation-dependent protein binding	4.7E − 04	Cytoskeletal anchor activity	7.2E − 03	Enzyme binding	3.0E − 04	Cell adhesion molecule binding	1.9E − 03
MF	GTPase regulator activity	7.6E − 03	Transcription coregulator binding	1.1E − 03	1-Phosphatidylinositol-4-phosphate 3-kinase activity	8.5E − 03	DNA-binding transcription factor binding	4.2E − 04	Beta-catenin binding	4.6E − 03
MF	Nucleoside-triphosphatase regulator activity	7.6E − 03	Proline-rich region binding	1.2E − 03	1-Phosphatidylinositol-3-kinase activity	8.5E − 03	Transcription coregulator activity	7.6E − 04	Phosphatidylinositol-3,4,5-trisphosphate binding	5.8E − 03
MF	Demethylase activity	8.4E − 03	Prostaglandin E receptor activity	5.8E − 03	Poly-pyrimidine tract binding	1.0E − 02	RNA polymerase II-specific DNA-binding transcription factor binding	8.5E − 04	Transferase activity, transferring phosphorus-containing groups	5.8E − 03
MF	bHLH transcription factor binding	9.2E − 03	Prostaglandin receptor activity	6.1E − 03	Ephrin receptor binding	1.1E − 02	Protein tyrosine kinase activity	9.8E − 04	Phosphatidylinositol-3,5-bisphosphate binding	7.0E − 03
MF	Histone demethylase activity	1.1E − 02	Transmembrane receptor protein tyrosine kinase activity	8.3E − 03	Branched-chain amino acid transmembrane transporter activity	1.2E − 02	Kinase activity	1.0E − 03	Cysteine-type endopeptidase regulator activity involved in apoptotic process	7.7E − 03
MF	Protein demethylase activity	1.1E − 02	Bicarbonate transmembrane transporter activity	9.2E − 03	Poly(U) RNA binding	1.2E − 02	ATP binding	1.9E − 03	Histone H3K9 methyltransferase activity	9.2E − 03
MF	RS domain binding	1.1E − 02	Transcription coactivator binding	9.8E − 03	Scaffold protein binding	1.3E − 02	Transcription coregulator binding	1.9E − 03	Ubiquitin-like protein transferase activity	9.2E − 03
MF	Transcription coregulator activity	1.3E − 02	Modification-dependent protein binding	1.3E − 02	Ubiquitin-like protein conjugating enzyme binding	1.4E − 02	Adenyl nucleotide binding	2.7E − 03	Cadherin binding	9.5E − 03

## Discussion

We performed the first systematic comparison of DNAm-health associations between two developmental timepoints (birth and childhood) on child outcomes spanning mental and physical domains. Our findings lend three important new insights: (1) effect sizes tend to be larger when DNAm is measured in childhood compared to at birth; (2) even though EWAS effect sizes consistently increase over time for all outcomes examined, this did not necessarily lead to more significant findings; (3) DNAm signals are largely distinct between timepoints, but they correlate across outcomes, indicating shared associations.

### Key finding 1: EWAS effect sizes increase over time for all child health outcomes

Our first key finding is that across *all* five outcomes, mean EWAS effect sizes increased over time: they were stronger in the cross-sectional childhood analyses as compared to the prospective birth analyses. This may be due to a number of reasons: (i) the temporal proximity of the cross-sectional EWASs may better reflect immediate causal effects of DNAm on an outcome; (ii) in addition to genetic and prenatal environmental factors captured by DNAm at birth, DNAm in childhood may also reflect the accumulation of relevant postnatal environmental exposures and genetic effects [11]; (iii) peripheral blood (in childhood) may be a more informative tissue than cord blood (at birth), e.g., due to tissue differences in cell-type composition or immune profile—although we do not find evidence of blood tissue-specific pathway enrichment; and (iv) there may be unmeasured confounding (e.g., lifestyle, allergens) and reverse causation in childhood, which is more likely to affect cross-sectional analyses than prospective analyses [26]. Indeed, Mendelian randomization studies suggest that for at least some sites, DNAm levels are a consequence, rather than a cause, of BMI [27, 28] or asthma [29]. While we can only speculate as to the most likely reason for the observed effect size increase, we can conclude that it is consistent for different outcomes, and to a comparable degree, hinting at potentially common driving factors.

### Key finding 2: Higher effect sizes ≠ more significant findings

While EWAS effect sizes robustly increased, this did not necessarily result in more significant findings, as the signal also became “noisier” with larger SE in childhood analyses. For BMI, effect size increases did correspond with statistical significance increases; however, for the other four outcomes, significance on average either remained the same or in the case of ADHD symptoms even decreased from birth to childhood. Statistical models were very similar between prospective and cross-sectional analyses and are unlikely to explain SE differences. Outcome definitions were identical and the same covariates were included, with the only exception of cell-type proportion estimates, to enable estimation using tissue-appropriate reference panels (i.e., cord or peripheral blood). The main difference was the predictor; i.e., when DNAm was assessed. The only study which applied different models was the EWAS of ADHD symptoms. Three of the nine participating cohorts used repeated ADHD measures for prospective models, but for cross-sectional analyses, we chose a single timepoint closest to DNAm measurement to maximize precision and power in prospective analyses, while ensuring concurrent assessment in cross-sectional analyses. The repeated measures design may have contributed to lower standard errors for the birth DNAm statistics. However, as this modeling difference only applied to the EWAS of ADHD symptoms, it is unlikely that using single vs repeated measure models can fully explain the lower standard errors observed for prospective vs cross-sectional analyses, since this pattern was also found for all other outcomes (which only relied on single measures), except BMI.

Three other plausible “culprits” for the noisier signal include sample size differences, between-study heterogeneity, and increasing DNAm variance with age. First, an imbalance in sample sizes (and associated power) between the birth and childhood EWASs could have led to differences in mean statistical significance. However, results remained largely consistent when restricting sample sizes to be equal between timepoints, ruling out this explanation. Second, we found that for all outcomes except GPF, between-study heterogeneity (systematic variability in effect sizes across cohorts) increased when DNAm was measured in childhood, potentially leading to more statistical uncertainty. Contributing factors may include (i) DNAm assessment age differences, which varied substantially less in EWAS analyses at birth (cohort differences in the order of days) compared to EWAS in childhood (age ranging from 5 to 17 years for asthma); and (ii) environmental differences between the included cohorts, which may cumulatively affect DNAm patterns (diet, pollutants, etc.), leading to more context-dependent associations in childhood. Importantly, however, between-study heterogeneity does not seem to fully account for increasing error in EWAS estimates over time. Indeed, when we re-ran meta-regression analyses only in ALSPAC, we found largely the same pattern of findings as the overall meta-analyses, meaning that sources of variability related to the use of multiple cohorts are unlikely to fully explain the observed temporal differences in the EWAS signal.

A third explanation relates to DNAm variance. Variance for most DNAm sites increases with age (on average increasing 1.26-fold per year from birth), with only a minority of DNAm sites showing significant decreases in variance [30]. It is likely that this increased variance reflects in part health-relevant variation, e.g., reflecting additional important postnatal exposures, resulting in increased effect sizes. At the same time, the increased variance likely also includes a substantial amount of variance unrelated to the studied health-related outcomes, increasing the noise of the DNAm estimates and lowering power.

In summary, our findings caution against the assumption that larger effect sizes in EWAS lead to the identification of more hits. Rather, they suggest that statistical power varies depending on factors such as the degree of uncertainty and study heterogeneity, the timing of DNAm assessment, and the potentially causal nature and direction of associations between DNAm and a given outcome.

### Key finding 3: epigenetic signals associated with child outcomes are time-specific and pleiotropic

Our analyses correlating EWAS estimates between timepoints reveal largely distinct association signals at birth versus in childhood: estimates at birth did not correlate with those in childhood—or only modestly in the case of ADHD symptoms. Whether this specificity in DNAm signals extends more broadly to other life stages, or DNAm associations become more stable and comparable after some developmental point cannot be inferred from the current data [10, 30]. These temporal differences raise the question of which DNAm assessment timepoint may be most relevant for health. For biomarker purposes, DNAm estimates from cross-sectional childhood analyses may explain the higher phenotypic variance, but at the cost of higher uncertainty of estimates. This may lead to less reliable methylation profile scores (MPS; akin to polygenic scores or PGS), which may also reflect consequences of a phenotype, and thus less useful for the prediction of later outcomes [31]. Our results caution that MPS computed from one DNAm timepoint may generalize poorly across development. Repeated assessments of DNAm and the combination of multiple age-specific scores may be needed to improve MPS performance, although specific guidelines are difficult to formulate. For instance, MPSs based on allergy-related EWAS performed similarly well when tested at both ages 6 and 10 years [32], but differences between birth and childhood methylation profiles are likely more impactful.

Surprisingly, the consistency of estimates across child outcomes was larger than between timepoints for the same outcome. Our analyses suggest that DNAm associations with ADHD symptoms, GPF, and asthma are to some degree shared. This is in line with previous studies pointing to phenotypic and genetic correlations [33–36] and may point towards early shared origins or network effects among the phenotypes reflected in the methylome. Enrichment analyses suggest that neural pathways may be involved in all tested health outcomes (particularly mental phenotypes) and may partly explain the observed correlations. However, the negative correlation between ADHD-related DNAm at birth/childhood and BMI-related DNAm in childhood is more perplexing. Children with ADHD are more likely to be overweight and vice versa [37, 38], and BMI and ADHD also show positive genetic correlations [34, 39]. The opposing correlation patterns may indicate that epigenetic risk mechanisms for ADHD are associated with lower BMI in childhood, but are overshadowed by (non-methylation) mechanisms causing positive phenotypic correlations. One such epigenetic mechanism may reflect mediation via ADHD medication use. ADHD-related DNAm levels could associate with increased probability of stimulant use. ADHD medications, such as methylphenidate, are in turn related to lower BMI [40]. It is unclear to which degree such mediation effects contribute to the negative correlation, given the low prevalence rates of stimulant medication use in these population-based cohorts [41], the small magnitude of reported effects of stimulant medicines on BMI [40], and the fact that medication use would not have affected DNAm levels at birth. Yet, it will be important to clarify these relationships in future studies, for example by performing an EWAS of ADHD medication (testing enrichment for medication-related DNAm patterns in EWAS of ADHD symptoms) or directly performing epigenome-wide mediation analyses.

### Study limitations and future research

The summary statistics-based approach enabled analysis of many outcomes and a large sample size, but also has limitations. The exact degree of sample overlap across timepoints and outcomes could not be explicitly modeled, though single cohort sensitivity analyses with largely overlapping samples did not alter conclusions. It was also not possible to model age at DNAm or phenotype assessment on an individual level and we had to rely on cohort averages. Another individual characteristic we cannot model with the given data is sex. Associations may differ depending on sex and affect childhood DNAm associations disproportionately, especially after puberty, as opposed to birth DNAm associations. Future studies with individual-level data should also study the impact of increasing DNAm variance on association estimates. Lastly, we could not perform formal epigenetic correlation tests and the regression coefficient correlations should therefore be interpreted as hypothesis-generating for future research.

With current study designs, it is also impossible to disentangle timing differences from tissue differences between cord and peripheral blood. Future studies are needed that examine different tissues at birth (to determine the specificity to cord blood as opposed to the neonatal period in general) as well as DNAm at multiple timepoints during childhood (to test if effect sizes change non-linearly across development) [10]. While our analyses provided important new insights into genome-wide trends, they were mostly underpowered to identify *specific* DNAm sites at a genome-wide level of significance; as such, larger studies are needed to reliably characterize epigenetic changes in associations for individual sites. Finally, expanding analyses to other outcomes should be pursued in future research.

## Conclusions

Overall, our results suggest developmentally-specific associations between DNAm and child health outcomes, when assessing DNAm at birth vs childhood. This implies that EWAS results from one timepoint are unlikely to generalize to another (at least based on birth vs childhood comparisons). This is a consequential finding, given that most research to date examines DNAm at a single assessment time-point. Longitudinal studies with repeated epigenetic assessments are direly needed to shed light on the dynamic relationship between DNAm, development and health, as well as to enable the creation of more reliable and generalizable epigenetic biomarkers. More broadly, this study underscores the importance of considering the time-varying nature of DNAm in epigenetic research and supports the potential existence of epigenetic “timing effects” on child health.

## Supplementary Information

Additional file 1. Table S1: Cohort sample sizes. Table S2: Number of cohorts overlapping. Table S3a: Absolute effect size percentiles. Table S3b: Number of DNAm sites with nominally significant change per effect direction pattern. Table S4: Association between DNA methylation either at birth or in childhood and child developmental outcomes. Table S5: Heterogeneity analyses. Table S6: Association between DNA methylation either at birth or in childhood and child developmental outcomes. Table S7: Enrichment ADHD. Table S8: Enrichment GPF. Table S9: Enrichment Sleep. Table S10: Enrichment BMI. Table S11: Enrichment Asthma

Additional file 2. Supplementary Figures.

Additional file 3. Cohort-specific information.

## Data Availability

Original analysis code and example data can be found at https://github.com/aneumann-science/epigenetic_timing_effects [42]. Full meta-analysis summary statistics can be downloaded at https://doi.org/10.5281/zenodo.10720466 [43].

## Abbreviations

DNAm: DNA methylation
ADHD: Attention-deficit/hyperactivity disorder
BMI: Body mass index
EWAS: Epigenome-wide association study
PACE: Pregnancy And Childhood Epigenetics Consortium
SD: Standard deviations
