# Supplementary material for: Epigenetic timing effects on child developmental outcomes: a longitudinal meta-regression of findings from the Pregnancy And Childhood Epigenetics Consortium
Source: Genome Med. 2025 Apr 14;17:39. doi: 10.1186/s13073-025-01451-7 (PMC11995515; doi:10.1186/s13073-025-01451-7)

Epigenetic timing effects on child developmental outcomes:
A longitudinal meta-regression of findings from the Pregnancy And Childhood Epigenetics Consortium

Additional File 2: Supplementary Figures

Fig. S1: Distribution of effect sizes and statistical significance for DNAm at birth and in childhood. Mean effect sizes (left column) and mean statistical significance (right column) across all tested autosomal DNAm sites per outcome (y-axis) and timepoint. Upper row displays results from analyses utilizing maximum available sample sizes. Lower row displays results from analyses with cohorts removed to achieve equal sample sizes at both timepoints. Effect size is given as absolute regression coefficient ($|\bar{\beta}|$), representing the difference in child health outcomes in SD between full or no methylation in the case of continuous outcomes (ADHD, general psychopathology, sleep duration and BMI), or log(odds ratio) for categorical outcomes (asthma diagnosis). Effect sizes are displayed on log-scale but y-axis units are in original units. Statistical significance is given as mean absolute Z-values and again displayed on a log-scale.

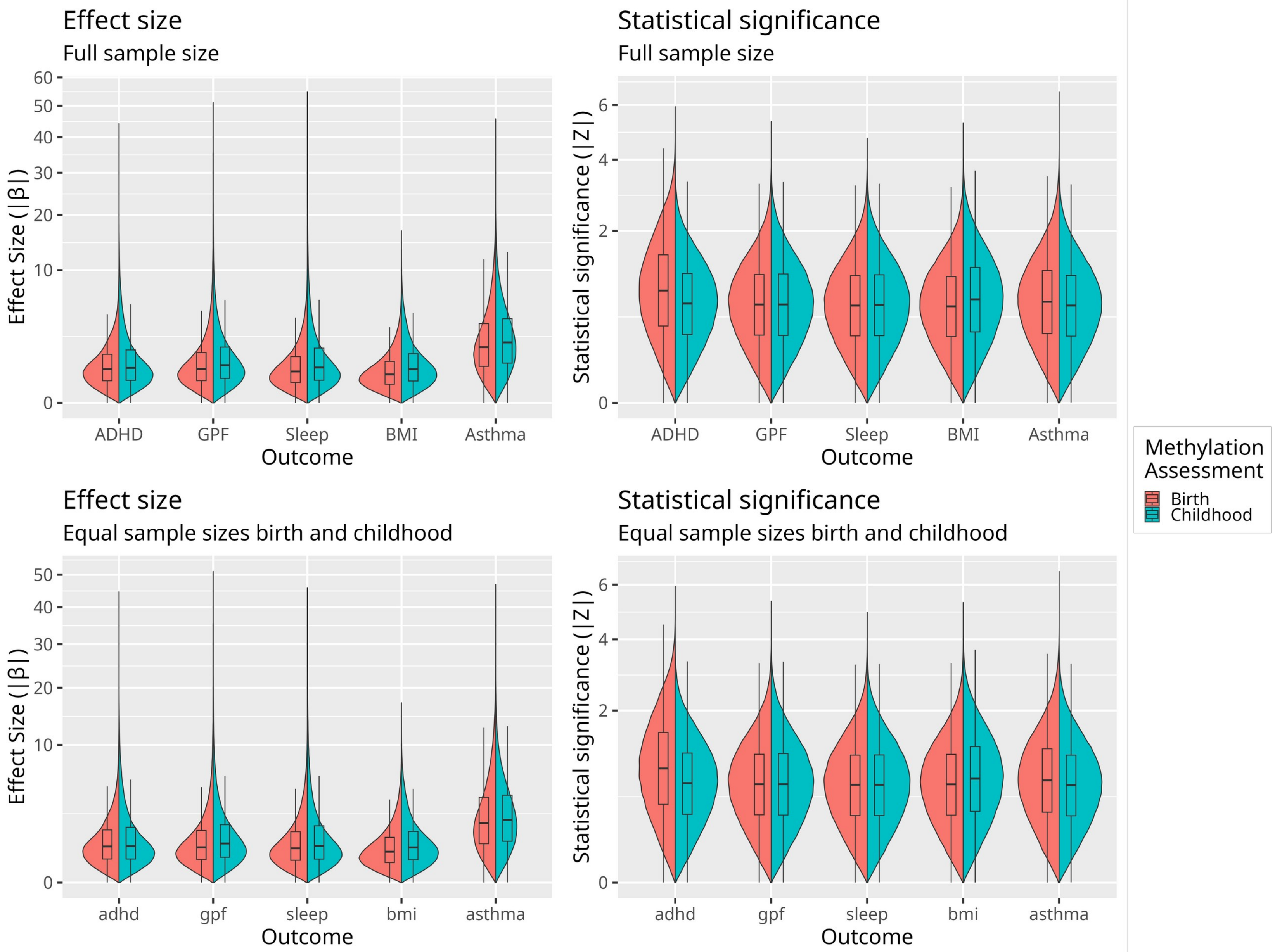
Effect size
Full sample size
Effect Size (|β|)
60
50
40
30
20
10
0
ADHD
GPF
Sleep
BMI
Asthma
Outcome
Statistical significance
Full sample size
Statistical significance (|Z|)
6
4
2
0
ADHD
GPF
Sleep
BMI
Asthma
Outcome
Methylation Assessment
Birth
Childhood
Effect size
Equal sample sizes birth and childhood
Effect Size (|β|)
50
40
30
20
10
0
adhd
gpf
sleep
bmi
asthma
Outcome
Statistical significance
Equal sample sizes birth and childhood
Statistical significance (|Z|)
6
4
2
0
adhd
gpf
sleep
bmi
asthma
Outcome

Fig. S2: Manhattan plot of DNAm associations at birth or in childhood. Manhattan plot displaying the -log$_{10}$ p-value (y-axis) for DNAm associations at birth (bottom plots) or in childhood (upper plots) per site (x-axis) per outcome. Red line indicates genome-wide significance (Bonferroni: 1/number of tested probes), yellow line represents suggestive threshold (1*10^{-5}). Bottom ideogram displays cytobands.

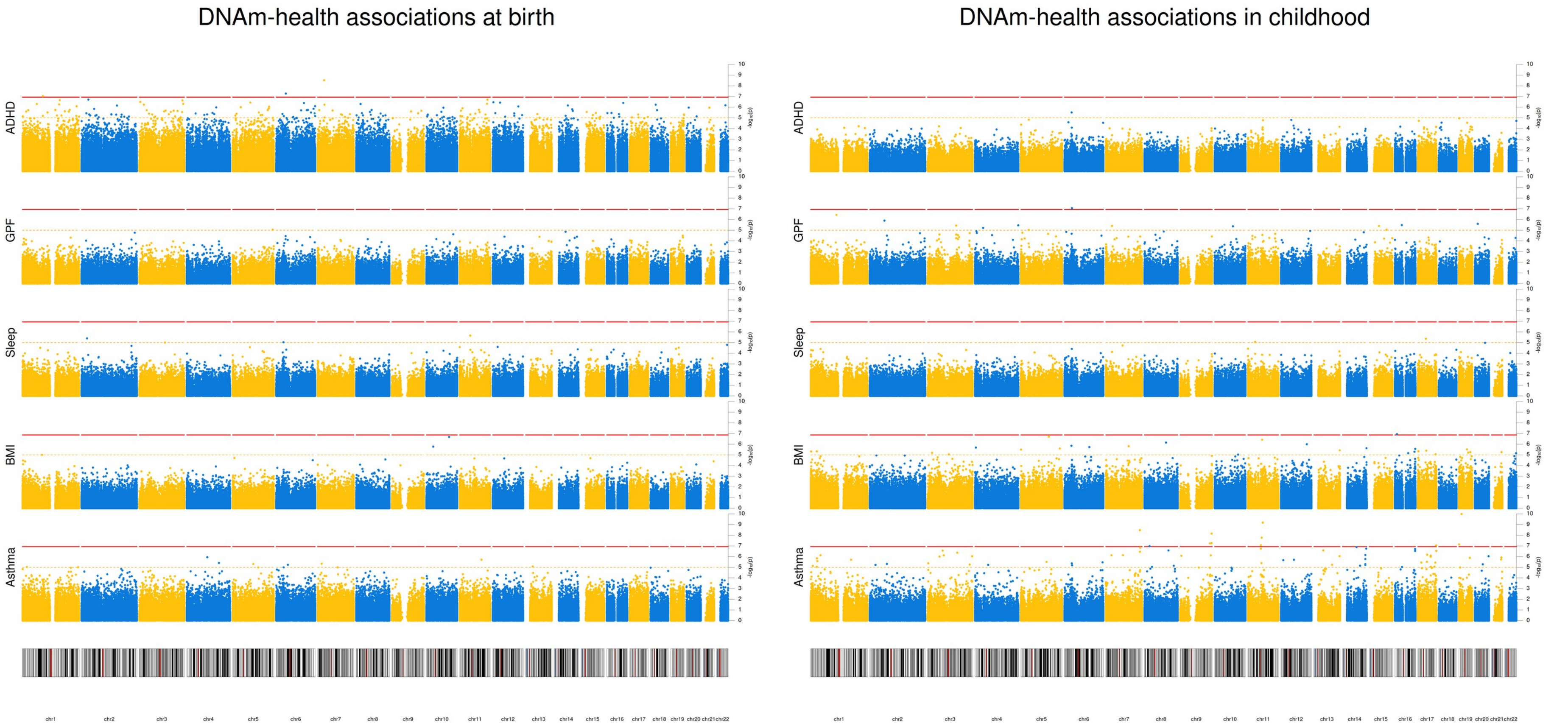

Fig. S3: Manhattan plot of association change. Manhattan plot displaying the -$\log_{10}$ p-value (y-axis) for change in DNAm association from birth to childhood per DNAm site (x-axis) per outcome. Red line indicates genome-wide significance (Bonferroni: 1/number of tested probes), yellow line represents suggestive threshold ($1*10^{-5}$). Bottom ideogram displays cytobands.

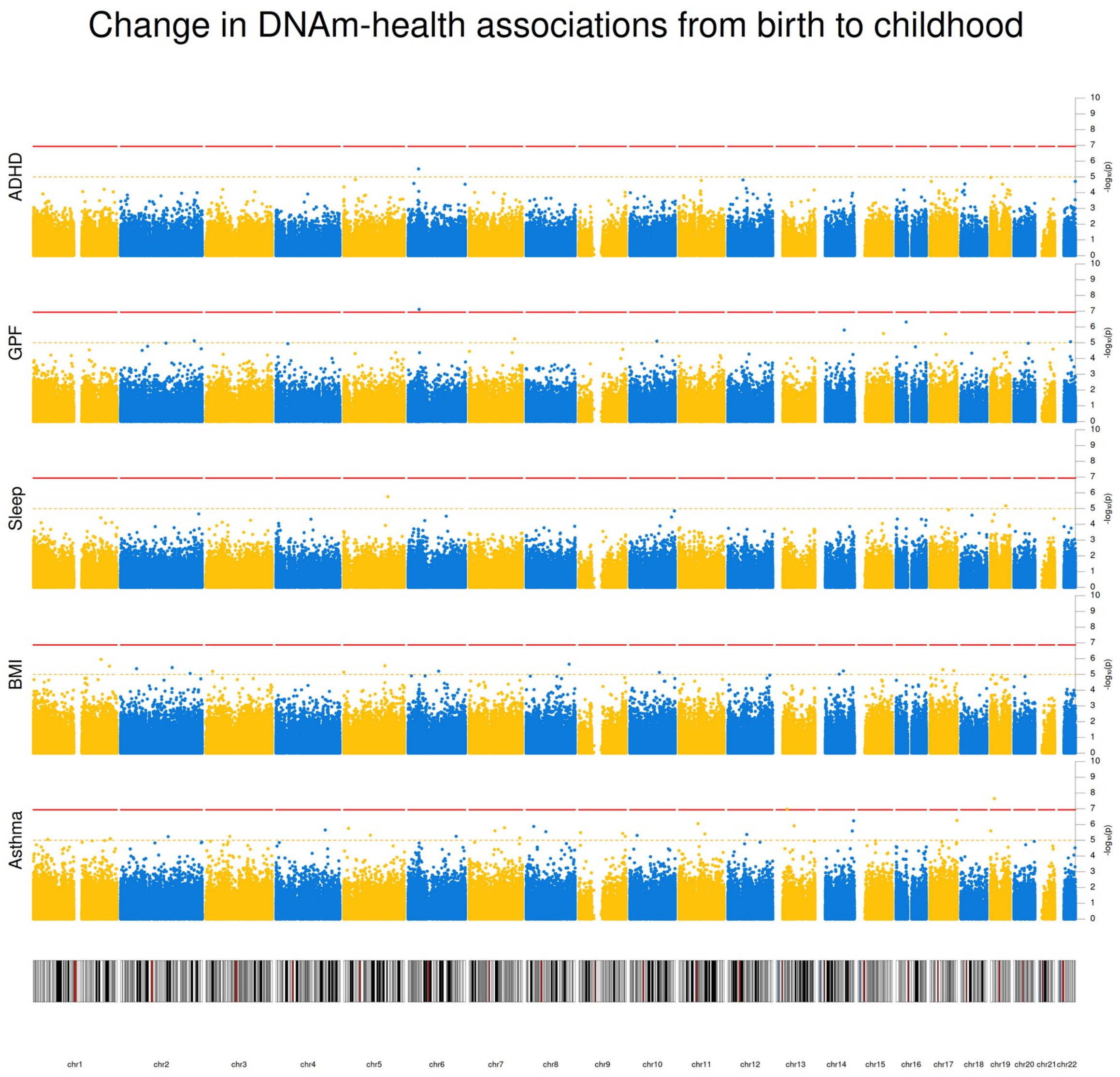

Fig. S4: Effect size change ratio across different p-value thresholds. Number of DNAm sites with increasing effect size divided by number of DNAm sites with decreasing effect size from birth to childhood (y-axis). Y-axis values higher than 1 indicate more DNAm sites with increasing effect size vs decreasing. DNAm is categorized as changing according to different p-value thresholds of change (-$\log_{10}$ p-values; x-axis). No thresholding based on statistical significance of change is applied on the left, but becomes increasingly stringent towards the right.

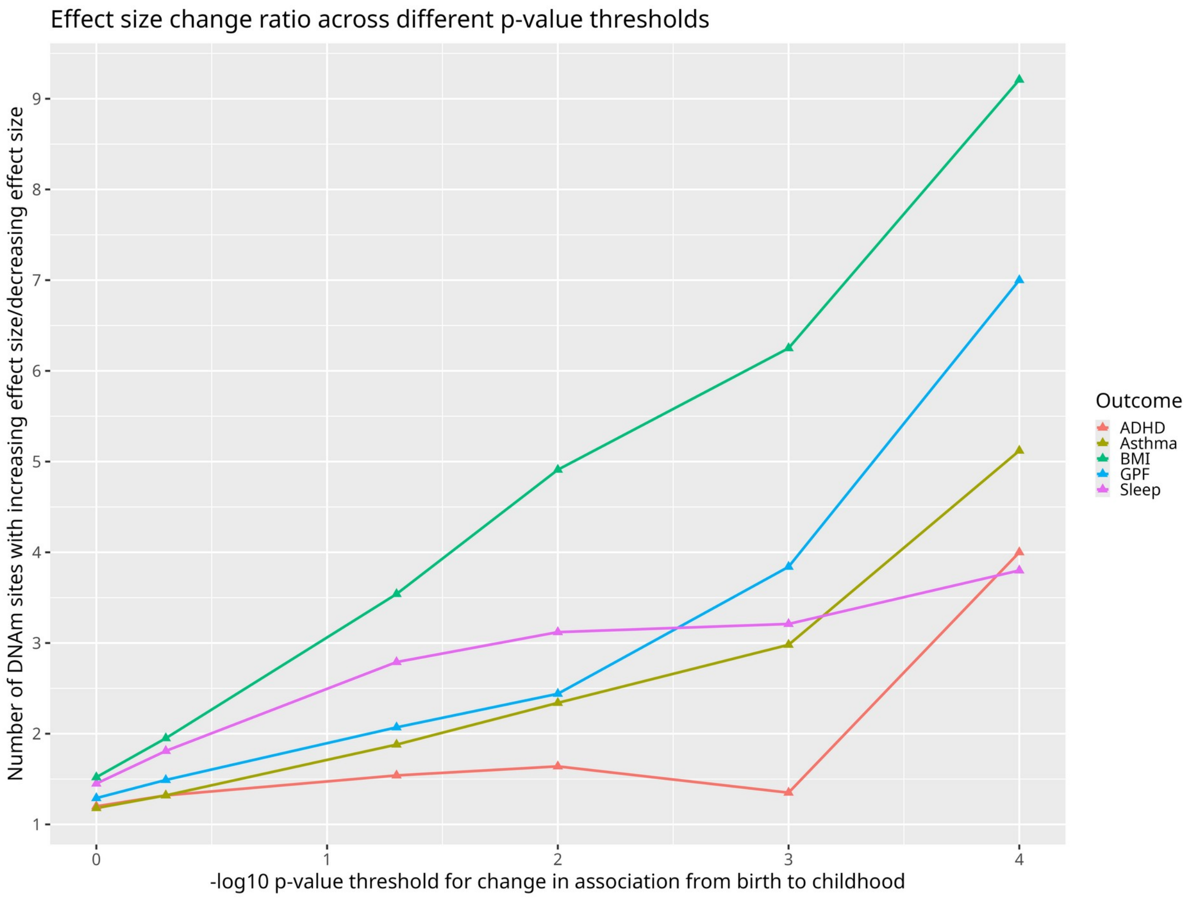

Fig. S5: Correlations between DNAm effects at birth and childhood and across outcomes in ASLPAC. This correlation matrix displays Spearman correlations between regression coefficients for DNAm at birth and childhood and across outcomes. Intensity of red represents higher positive correlations and blue lower negative correlations. Estimates are based on ALSPAC cohort only.

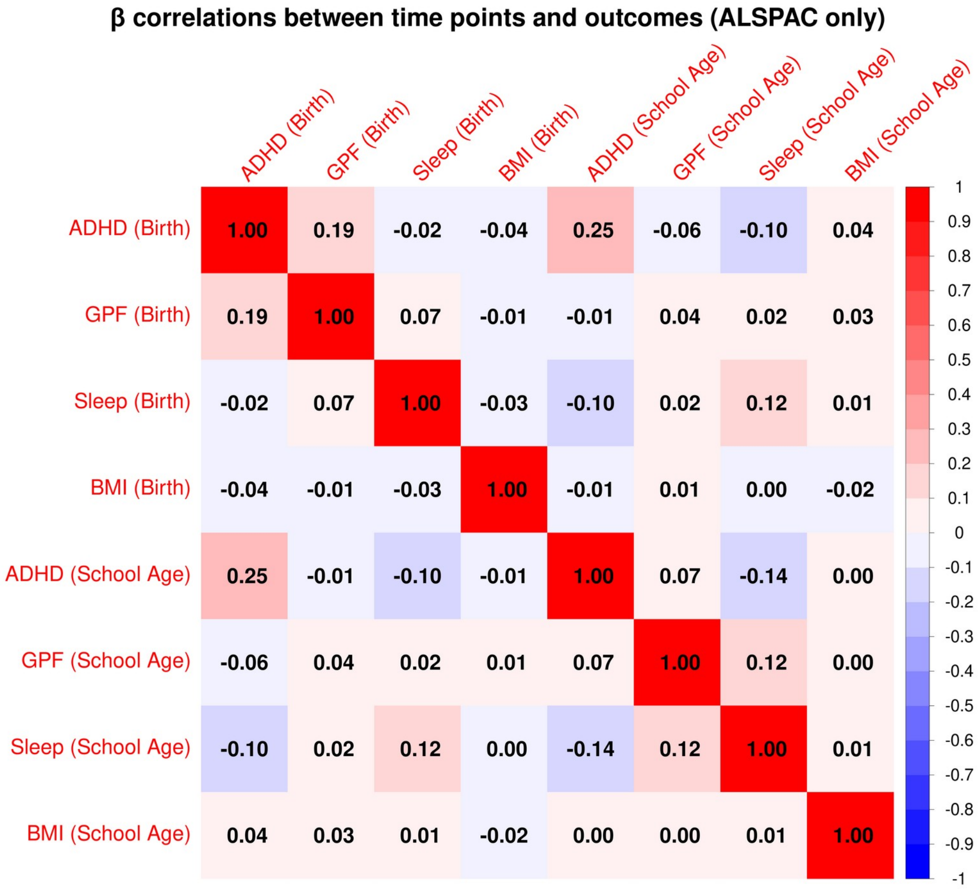

Supplement: Supplementary file 2 — Additional file 2. Supplementary Figures. [file 13073_2025_1451_MOESM2_ESM.pdf]
